# Nrf2/HO-1 mediates the neuroprotective effects of pramipexole by attenuating oxidative damage and mitochondrial perturbation after traumatic brain injury in rats

**DOI:** 10.1242/dmm.045021

**Published:** 2020-08-19

**Authors:** Mohd. Salman, Heena Tabassum, Suhel Parvez

**Affiliations:** 1 Department of Medical Elementology and Toxicology, School of Chemical and Life Sciences, Jamia Hamdard, New Delhi 110062, India; 2 Division of Basic Medical Sciences, Indian Council of Medical Research, Ministry of Health and Family Welfare, Government of India, V. Ramalingaswamy Bhawan, New Delhi 110 029, India

**Keywords:** Traumatic brain injury, Oxidative damage, Mitochondrial dysfunction, Nrf2, Pramipexole, Neuroprotection

## Abstract

Pramipexole (PPX), a D2-like receptor agonist, is generally used in the treatment of Parkinson's disease and restless leg syndrome. Its neuroprotective effects have been shown against various neurological disorders. Recent research work has demonstrated that PPX exerts neuroprotection through mitochondria. However, the neuromodulator-related effects of PPX against traumatic brain injury (TBI) remain unexplored. The present study, therefore, investigated the mechanism of neuroprotection by PPX against oxidative stress, mitochondrial dysfunction and neuronal damage following TBI in rats. We hypothesized that the neuroprotection by PPX in TBI-subjected rats might involve activation of the Nrf2/HO-1 (also known as Nfe2l2/Hmox1) signaling pathway. PPX was injected intraperitoneally (0.25 mg/kg body weight and 1.0 mg/kg body weight) at different time intervals post-TBI. Several neurobehavioral parameters were assessed at 48 h post-TBI, and the brain was isolated for molecular and biochemical analysis. The results demonstrated that PPX treatment significantly improved the behavioral deficits, decreased the lipid peroxidation rate, increased glutathione levels and decreased 4-hydroxynonenal levels in TBI-subjected rats. PPX also increased the activities of glutathione peroxidase and superoxide dismutase enzymes. In addition, PPX treatment inhibited mitochondrial reactive oxygen species production, restored mitochondrial membrane potential and increased ATP levels after a TBI. Further, PPX treatment reduced the Bax/Bcl2 ratio and translocation of Bax to mitochondria and cytochrome-c to the cytosol. Finally, PPX treatment greatly accelerated the translocation of Nrf2 to the nucleus and upregulated HO-1 protein expression. We conclude that the neuroprotective effects of PPX are mediated by activation of the Nrf2/HO-1 signaling pathway following TBI.

This article has an associated First Person interview with the first author of the paper.

## Abstract (precis)

**Editor's choice:** We administered pramipexole (PPX) to rats after a traumatic brain injury (TBI) and evaluated various parameters, which indicate that the neuroprotective effects of PPX are mediated by activation of the Nrf2/HO-1 signaling pathway following TBI.

## INTRODUCTION

A traumatic brain injury (TBI) can have severely detrimental effects on human health. It is associated with difficulties in communication and behavioral impairments, which, in turn, contribute to socioeconomic problems for people of all ages throughout the world (Bruns and Hauser, 2003). During brain trauma, the brain tissue sustains damage from external mechanical forces. This, in turn, triggers compound molecular, neurochemical and histopathological alterations in the injured tissue. These changes can subsequently lead to deleterious side effects, and ultimately lead to neuronal cell death and dysfunction, permanent tissue damage and long-term neurological impairments (Mckee and Daneshvar, 2015). TBI causes two different types of injuries: primary injury (also known as mechanical injury), a prompt and destructive non-irreversible mechanical injury that disrupts the brain vasculature leading to hemorrhage, edema and ischemic condition within the brain, and secondary injury, which is initiated shortly after the first phase. It is also known as non-mechanical injury and can result in excitotoxicity, neuroinflammation, oxidative damage, calcium overload, pro-apoptotic gene activation, organelle dysfunction and neuronal apoptosis (Dobrachinski et al., 2017; Ng and Lee, 2019; Caglayan et al., 2019). Currently, there are no pharmacologically active chemicals that can attenuate TBI-induced oxidative damage and mitochondrial perturbation, and promote functional recovery following a TBI.

Experimental evidence indicates that mitochondria are the power plant of a cell. Mitochondria provide energy via oxidative phosphorylation and enable neurons to maintain proper function as well as control various pathological processes (Zhang et al., 2017a). Mitochondria are also the primary hub of free radical generation, including reactive oxygen species (ROS) and reactive nitrogen species (RNS). Excess ROS/RNS production increases protein damage, deoxyribonucleic acid damage and lipid peroxidation, halts cellular energy generation and induces metabolic failure, further causing neuronal cell death and dysfunction (Zorov et al., 2014). A growing number of basic and clinical experiments have demonstrated that mitochondrial injury/dysfunction play a crucial role in neuronal survival and death (Hill et al., 2017). However, mitochondrial biogenesis is a dynamically regulated progression mechanism. It can improve mitochondrial function, maintain mitochondrial quality control and exert neuroprotection by enhancing neuronal function against mitochondrial perturbation owing to the pathological factors of TBI (Li et al., 2017).

Nuclear factor erythroid 2-related factor-2 (Nrf2; also known as Nfe2l2) is a transcription factor and important upregulator of the endogenous cell defense mechanism in response to oxidative damage and is involved in restoration of intracellular homeostasis (Li et al., 2019b). Nrf2 is a basic leucine zipper (bZIP) cytoplasmic protein, which translocates into the nucleus under oxidative stress. It also binds to specific deoxyribonucleic acid antioxidant response elements (AREs) to initiate the transcription of cytoprotective genes such as heme oxygenase-1 (*HO-1*; also known as *Hmox1*), superoxide dismutase family members (SODs) and glutathione reductase (*Gsr*) in the nucleus (Chen et al., 2018). It has been reported that an increased level of Nrf2 exerts neuroprotective effects in TBI (Caglayan et al., 2019) and neurodegenerative diseases due to amelioration of oxidative damage (Xie et al., 2019). Thus, modulation of Nrf2 is a potential strategy to attenuate TBI-induced oxidative damage and mitochondrial dysfunction.

Pramipexole (S)-2-amino-4,5,6,7-tetrahydro-6-propylamino benzothiazole di-hydrochloride monohydrate (PPX) is a dopamine (D3/2) receptor agonist. The US Food and Drug Administration (FDA) has approved it as a drug used for the treatment of Parkinson's disease and restless leg syndrome (Pich and Collo, 2015; Benbir and Guilleminault, 2006). It is reported that PPX exerts neuroprotective effects by reducing oxidative damage, mitochondrial dysfunction and apoptosis, owing to its role as an inhibitor or scavenger of free radicals, and blocks mitochondrial permeability transition pores (mtPTPs) through the inhibition of depolarizing membrane currents in dysfunctional mitochondria in various models (Inden et al., 2009; Sayeed et al., 2006; Sadeghi et al., 2017). PPX inhibits the early brain injury after subarachnoid hemorrhage (Ma et al., 2016) and significantly reduces lipid peroxidation in a rat model of Parkinson's disease (Zou et al., 2000). Recently, our laboratory reported that PPX post-treatment provides mitochondrial-mediated neuroprotection via closing mtPTPs in an ischemic stroke model (Andrabi et al., 2019). Furthermore, pre-treatment of PPX protects PC12 cells treated with hydrogen peroxide through the activation of mitogen-activated protein (Fujita et al., 2006).

Therefore, the present study investigated the neuromodulatory effects of PPX after a TBI. We examined the effects of PPX on TBI-induced oxidative damage and mitochondrial injury in experimental rats. In addition, we explored the neuroprotective effects of PPX on mitochondrial perturbation and its possible ameliorative mechanisms in the brain after a TBI. To the best of our knowledge, this is the first study on TBI that shows that PPX treatment ameliorates oxidative damage and mitochondrial dysfunction, and promotes an antioxidant defense system through activation of the Nrf2/HO-1 signaling pathway. Findings from the study provide new insights into the potential therapeutic value of PPX for TBI.

## RESULTS

### Effects of PPX on neurobehavioral deficits in TBI rats

**Fig. 1.**
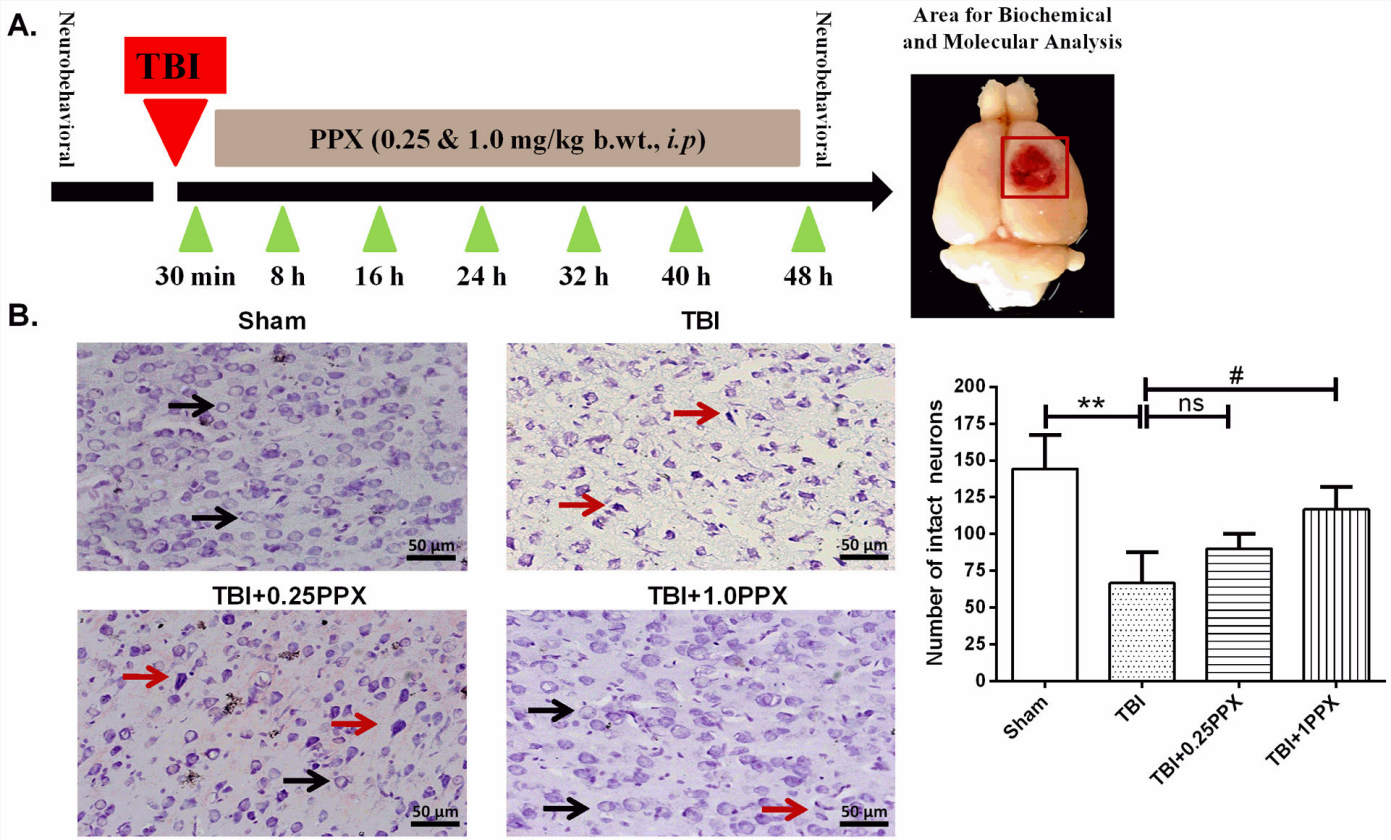
**PPX administration reduces neuronal damage after TBI.** (A) Timeline of the experimental treatment regimen used in this study (left). The area of the rat brain used for molecular and biochemical analysis is also shown (right). (B) Effects of PPX treatment on histological deficits in TBI rats. The arrows indicate the morphology of normal (black) and damaged (red) neurons in cortical rat brain tissue. In the sham group, neurons were intact with a clear border and a light stain. In the TBI group, the number of intact neurons was decreased, and the number of damaged neurons demonstrating cell shrinkage and dark staining was increased. PPX treatment increased the number of intact neurons. **P<0.01 compared with the sham group; ^#^*P*<0.05 compared with the TBI group; ns, not significant (one-way ANOVA with Tukey's multiple comparisons test). Data are presented as mean±s.d. (*n*=3). Magnification at 20×.

**Fig. 2.**
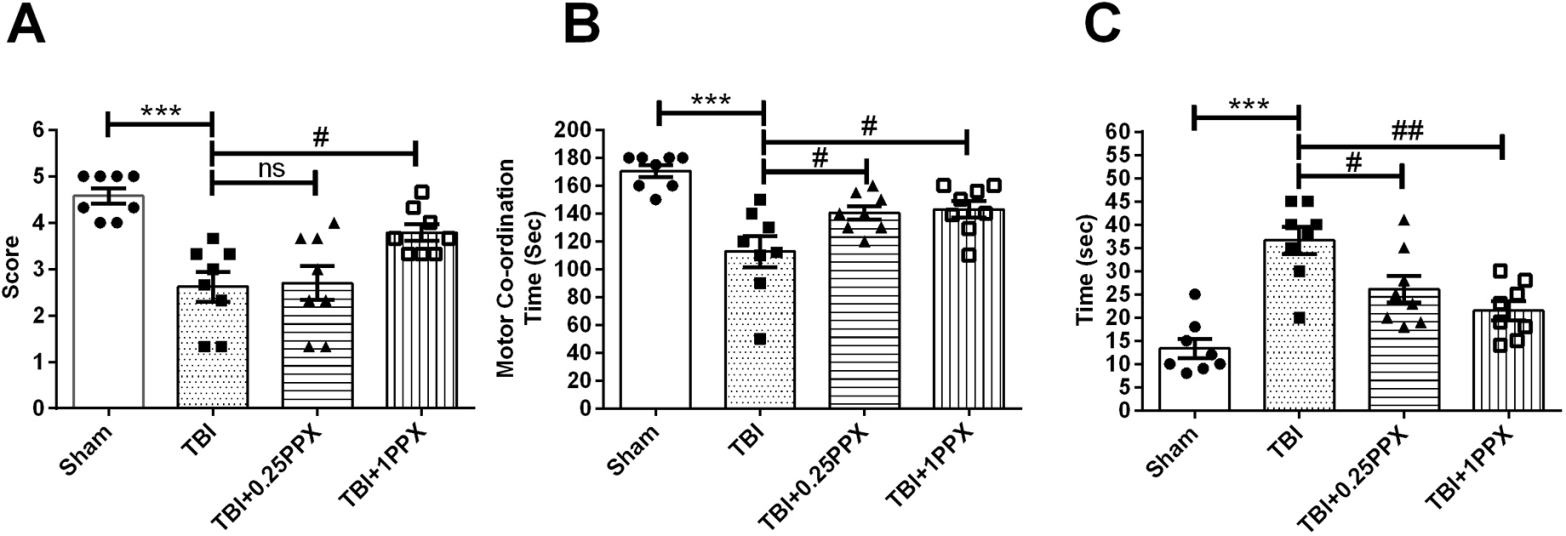
**PPX administration ameliorates neurological impairments.** (A) Grip test: TBI rats had significantly lower grip test scores than rats in the sham group. PPX-treated rats showed a significant increase in grip test scores compared with the TBI rats. (B) Rotarod test: TBI rats had significantly lower motor test performance compared with the sham rats. PPX-treated rats showed a significant improvement in rotometric performance compared with the TBI rats. (C) Beam balance test: TBI rats spent significantly more time crossing the narrow beam than did the sham rats. PPX-treated rats took significantly less time crossing the narrow beam than did the TBI rats. ***P<0.001 compared with the sham group; ^#^*P*<0.05 and ^##^*P*<0.01 compared with the TBI group; ns, not significant (one-way ANOVA with Tukey's multiple comparisons test). Data are presented as mean±s.e.m. (*n*=8).

Fig. 1A shows a timeline of the experimental treatment regimen used in this study. To investigate the neuroprotective effects of PPX after TBI, we evaluated behavioral activities using tests such as the grip, rotarod and beam balance tests in four groups of experimental rats: sham, TBI, TBI+PPX post-treatment [0.25 mg/kg body weight (b.wt.)] and TBI+PPX post-treatment (1.0 mg/kg b.wt.). First, we evaluated the grip test and found that the TBI-subjected rats showed a significant decrease [*F*_(3,28)_=11.9, *P*<0.0001] in the mean grip score compared with the sham rats. However, post-treatment with PPX at 1.0 mg/kg b.wt. significantly increased (*P*<0.05) the mean grip score in the TBI rats. There was no significant change in mean grip score at a low dose of PPX (Fig. 2A). Second, we evaluated performance in the rotarod test and found that the TBI rats spent significantly reduced [*F*_(3,28)_=11.0, *P*<0.0001] time on the rotating rod compared with the sham rats. PPX post-treatment significantly increased [0.25 mg/kg b.wt. and 1.0 mg/kg b.wt. (*P*<0.05 for both doses)] the time spent on the rotarod in the TBI rats (Fig. 2B). Finally, we evaluated the beam balance test, and observed that the TBI-subjected rats were unable to cross the narrow beam and took more time [*F*_(3,28)_=14.7, *P*<0.0001] to reach the box than did the sham rats. PPX post-treatment [0.25 mg/kg b.wt. (*P*<0.05) and 1.0 mg/kg b.wt. (*P*<0.01)] significantly improved the beam balance test performance, and PPX-treated rats were able to cross the beam in a shorter time compared with the TBI rats (Fig. 2C).

### Protective effects of PPX on oxidative stress biomarkers in TBI rats

**Fig. 3.**
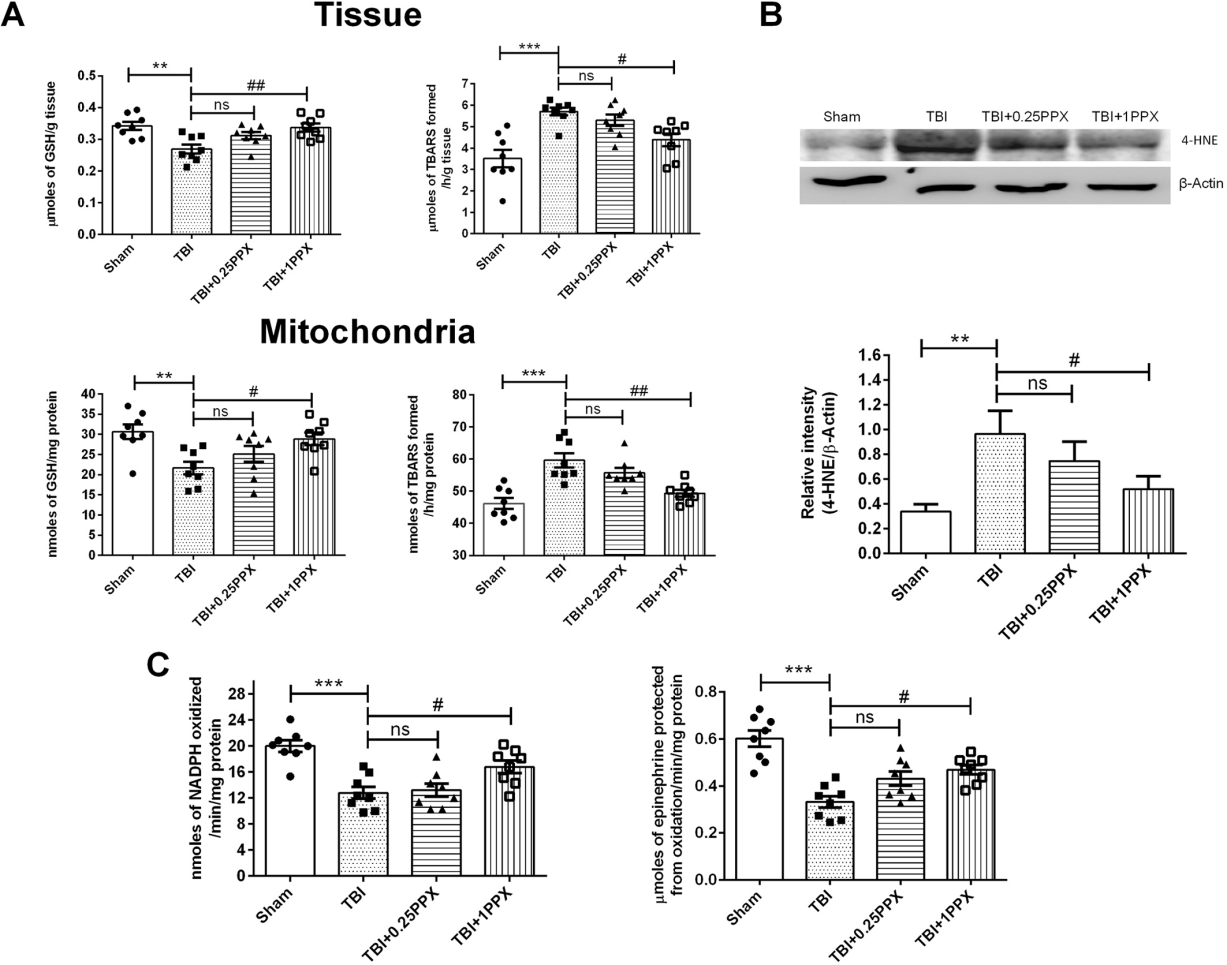
**PPX administration attenuates oxidative stress after TBI.** (A) In the TBI rats, LPO rate was significantly increased in the tissue (top) as well as mitochondrial (bottom) fraction. Rats treated with 1.0 mg/kg b.wt. PPX demonstrated significantly reduced LPO rate compared with the TBI rats. The level of GSH was significantly decreased in tissue and isolated mitochondria in the TBI rats; PPX treatment restored the GSH level in both the tissue and mitochondrial fraction. (B) In TBI rats, 4-HNE was significantly increased, indicating a higher LPO rate compared with that of the sham rats. Post-treatment with 1.0 mg/kg b.wt. significantly reduced the level of 4-HNE in the TBI-subjected rats. (C) The activities of antioxidant enzymes GPx (left) and SOD1 (right) were significantly lower in the TBI rats than in the sham rats. Post-treatment with PPX increased the activity of GPx and SOD1, significantly at 1.0 mg/kg b.wt. ***P<0.001 and **P<0.01 compared with the sham group; ^#^*P*<0.05 and ^##^*P*<0.01 compared with the TBI group; ns, not significant (one-way ANOVA with Tukey's multiple comparisons test). Data are presented as mean±s.e.m. (*n*=8). TBARS, thiobarbituric acid reactive substances.

To evaluate the antioxidant effects of PPX against focal TBI, we evaluated biochemical parameters such as lipid peroxidation (LPO), glutathione (GSH), glutathione peroxidase (GPx) and superoxide dismutase 1 (SOD1). As shown in Fig. 3A, our experimental results showed a significant decrease in GSH levels [*F*_(3,28)_=6.82, *P*=0.0014] and an increased LPO rate [measured as µmol/nmol thiobarbituric acid reactive substances (TBARS) formed/h/g tissue or mg protein] [*F*_(3,28)_=10.9, *P*=0.0001] in penumbra tissue as well as isolated mitochondria [GSH, *F*_(3,28)_=5.35, *P*=0.0049; LPO, *F*_(3,28)_=13, *P*=0.0001] of rats in the TBI group compared with those in the sham group. Post-treatment with PPX (1.0 mg/kg b.wt.) significantly restored the GSH levels [tissue (*P*<0.01) and mitochondria (*P*<0.05)] and reduced the LPO rate [tissue (*P*<0.05) and mitochondria (*P*<0.01)] in brain tissue and isolated mitochondria in the TBI-subjected rats. We also investigated the levels of the carbonyl-containing molecule 4-hydroxynonenal (4-HNE), which is the end product of lipid peroxidation. We observed significantly upregulated (*P*<0.01) 4-HNE levels in the TBI group compared with the sham group. PPX administration (1.0 mg/kg b.wt.) significantly reduced (*P*<0.05) the levels of 4-HNE in the TBI rats (Fig. 3B). Next, we analyzed the activities of GPx and SOD1 enzymes (as shown by nmol NADPH oxidized/min/mg protein and μmol epinephrine protected from oxidation/min/mg protein, respectively) in injured tissue as shown in Fig. 3C. Rats with focal TBI demonstrated inhibited activity of GPx and SOD1 enzymes [*F*_(3,28)_=12.8, *P*=0.0001 and *F*_(3,28)_=15.8, *P*=0.0001, respectively] compared with the sham rats. Post-administration of PPX (1.0 mg/kg b.wt.) significantly increased (*P*<0.05) the activity of both enzymes in the TBI rats. However, low-dose PPX (0.25 mg/kg b.wt.) failed to exert significant effects.

### Effects of PPX treatment on mitochondrial ROS in TBI rats

**Fig. 4.**
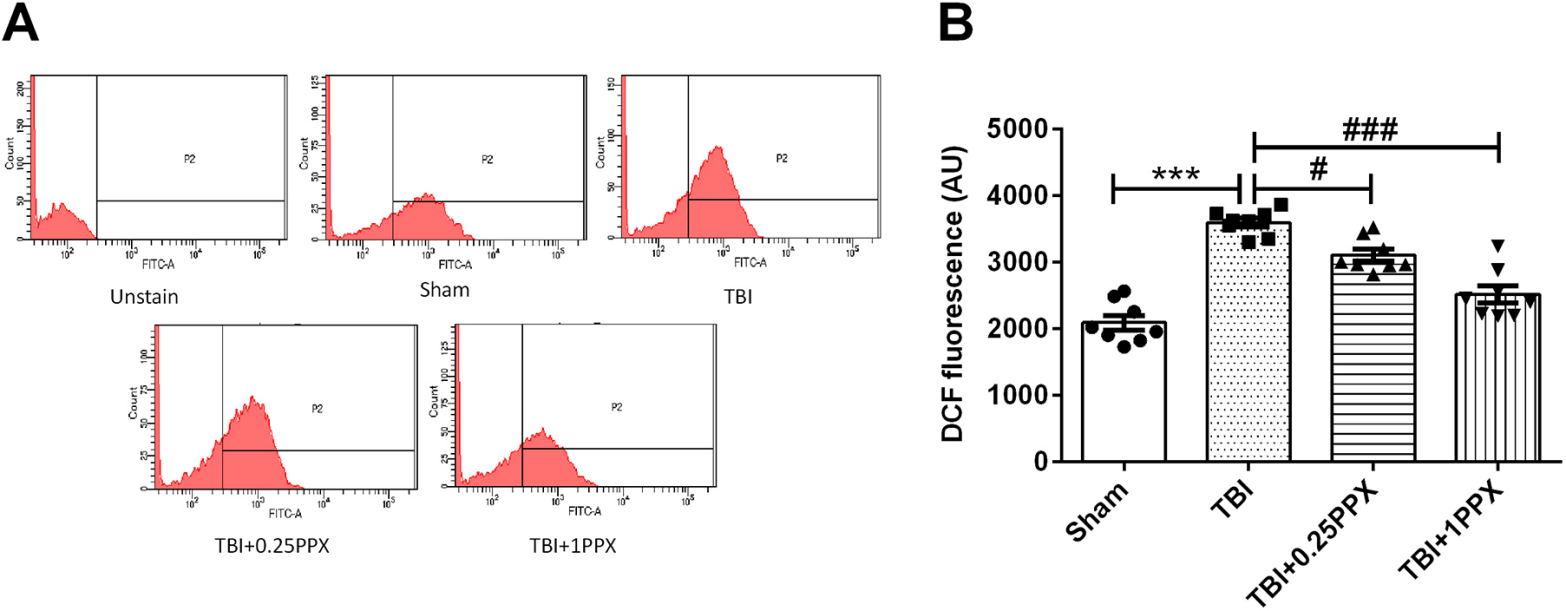
**PPX administration reduces mitochondrial ROS production.** (A) DCF fluorescence in mitochondria in the sham, TBI, TBI+0.25PPX and TBI+1PPX groups. (B) Mean fluorescence intensity (MFI) of DCF. ***P<0.001 compared with the sham group; ^#^*P*<0.05 and ^###^*P*<0.001 compared with the TBI group (one-way ANOVA with Tukey's multiple comparisons test). Data are presented as mean±s.e.m. (*n*=8). AU, arbitrary units.

Mitochondrial ROS production was evaluated as intensity of dichlorofluorescein (DCF) fluorescence. TBI induced a significant increase [*F*_(3,28)_=41.6, *P*=0.0001] in ROS production, as indicated by the increase in DCF fluorescence in the TBI group compared with the sham group. Post-treatment with PPX significantly reduced [0.25 mg/kg b.wt. (*P*<0.05) and 1.0 mg/kg b.wt. (*P*<0.001)] ROS production in the TBI rats (Fig. 4A,B).

### Effects of PPX treatment on mitochondrial membrane potential in TBI rats

**Fig. 5.**
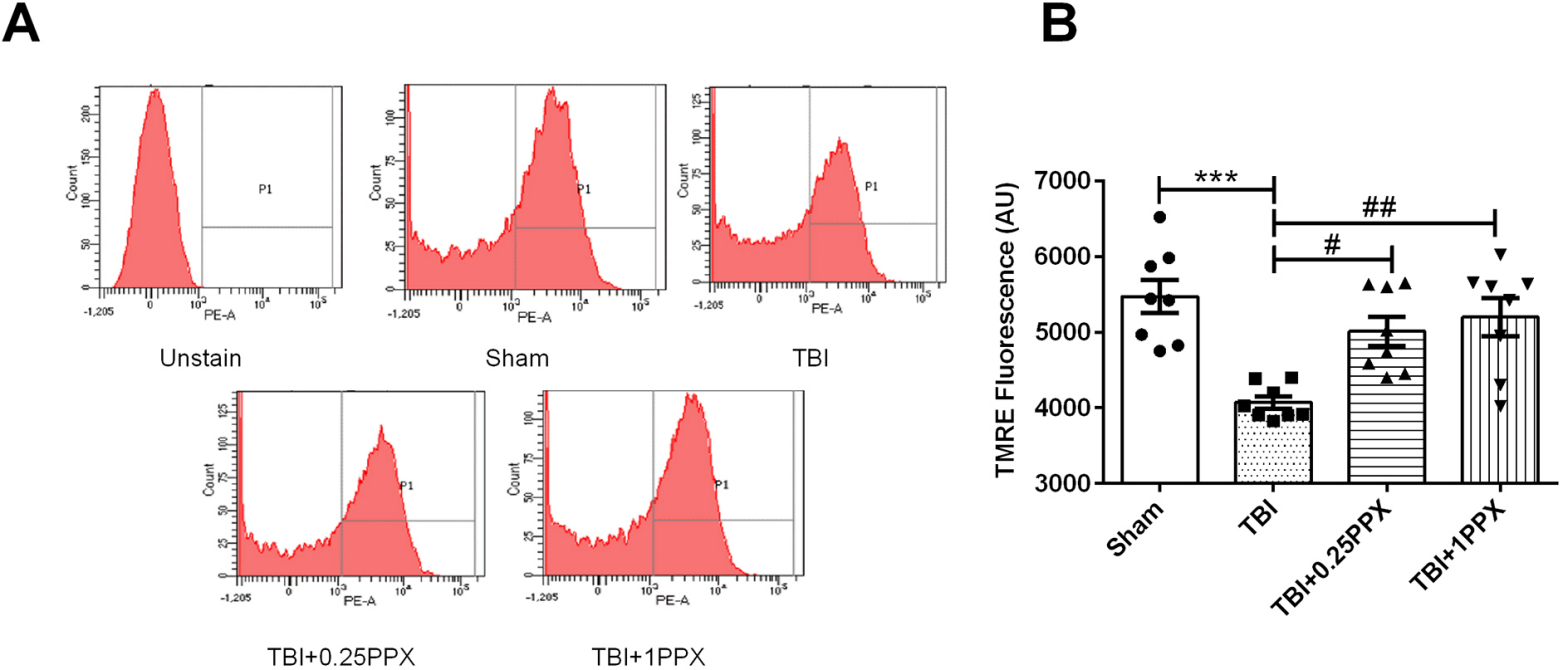
**PPX administration modulates the mitochondrial membrane potential.** (A) TMRE fluorescence in mitochondria in the sham, TBI, TBI+0.25PPX, and TBI+1PPX groups. (B) MFI of TMRE. ***P<0.001 compared with the sham group; ^#^*P*<0.05 and ^##^*P*<0.01 compared with the TBI group (one-way ANOVA with Tukey's multiple comparisons test). Data are presented as mean±s.e.m. (*n*=8). AU, arbitrary units.

With regard to the effects of TBI on mitochondrial membrane potential (Δψm), we found a significant decrease [*F*_(3,28)_=9.55, *P*=0.0002] in the mean fluorescence intensity (MFI) of tetramethylrhodamine ethyl ester (TMRE) dye in the TBI group compared with the sham group. This finding, as mentioned above, is indicative of mitochondrial dysfunction. However, PPX-treated rats showed significantly increased Δψm [0.25 mg/kg b.wt. (*P*<0.05) and 1.0 mg/kg b.wt. (*P*<0.01)] compared with the TBI rats, as shown in Fig. 5A,B.

### Effects of PPX treatment on Bax/Bcl2 ratio, cytochrome-c release and Bax translocation in TBI rats

**Fig. 6.**
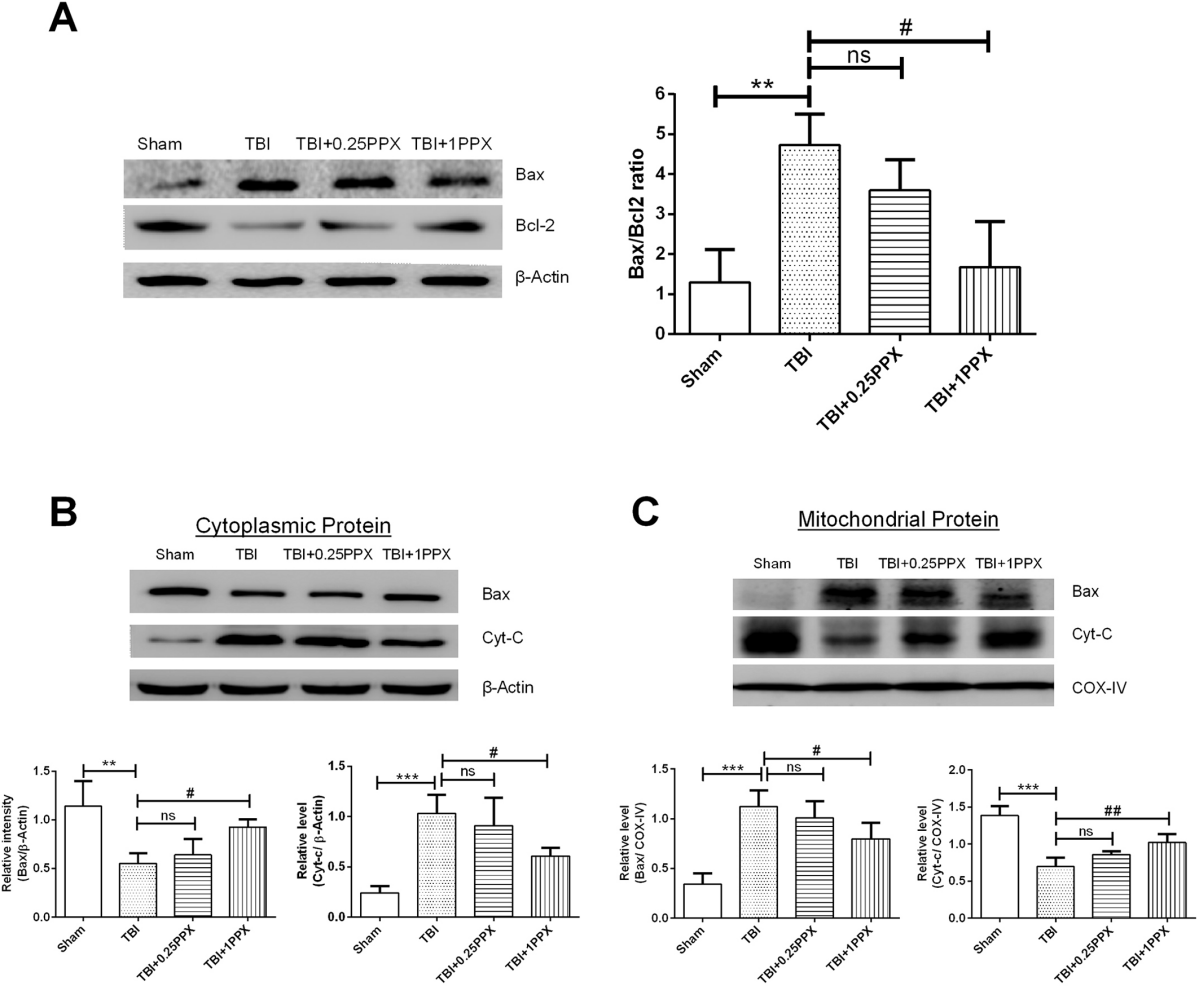
**PPX administration significantly downregulates the Bax/Bcl2 ratio and inhibits cyt-c release and Bax translocation.** (A-C) Representative immunoblot intensity indicates the Bax/Bcl2 ratio (A), and cytosolic (B) and mitochondrial (C) Bax and cyt-c. The intensity of the targeted protein bands was normalized to β-actin and COX-IV (also known as COX4I1). ***P<0.001 and **P<0.01 compared with the sham group; ^#^*P*<0.05 and ^##^*P*<0.01 compared with the TBI group; ns, not significant (one-way ANOVA with Tukey's multiple comparisons test). Data are presented as mean±s.d. (*n*=4).

The ratio of pro-apoptotic protein Bax and anti-apoptotic protein Bcl2 can be used as a biomarker of the apoptosis index. In the TBI group, we found a significant increase in Bax expression and decrease in Bcl2 expression compared with the sham group. In addition, PPX administration significantly reduced the Bax/Bcl2 ratio (Fig. 6A). We also investigated mitochondrial-dependent apoptosis proteins such as Bax and cytochrome-c (cyt-c; also known as Cycs) to explore the mechanism of neuroprotection of PPX against TBI. Following a TBI, Bax translocates to mitochondria and bonds with the outer mitochondrial membrane (Cheng et al., 2012). It also increases the permeability of mitochondria (Cheng et al., 2012). The brain tissue of the TBI rats demonstrated significantly higher mitochondrial Bax expression (*P*<0.001) and lower cytoplasmic Bax expression (*P*<0.01) compared with that of the sham rats. In contrast, mitochondrial and cytoplasmic cyt-c levels decreased (*P*<0.001) and increased (*P*<0.001), respectively, in the TBI group compared with the sham group (Fig. 6B,C). These alterations were reversed by PPX (1.0 mg/kg b.wt.) treatment, which significantly inhibited the mitochondrial translocation of Bax (*P*<0.05) and subsequent leakage of cyt-c (*P*<0.05) into the cytoplasm in rats post-TBI. There were no significant changes observed at a low dosage of PPX. Thus, these results confirmed that PPX reduced mitochondrial-mediated neuronal death after TBI.

### Effects of PPX treatment on ATP content in TBI rats

**Fig. 7.**
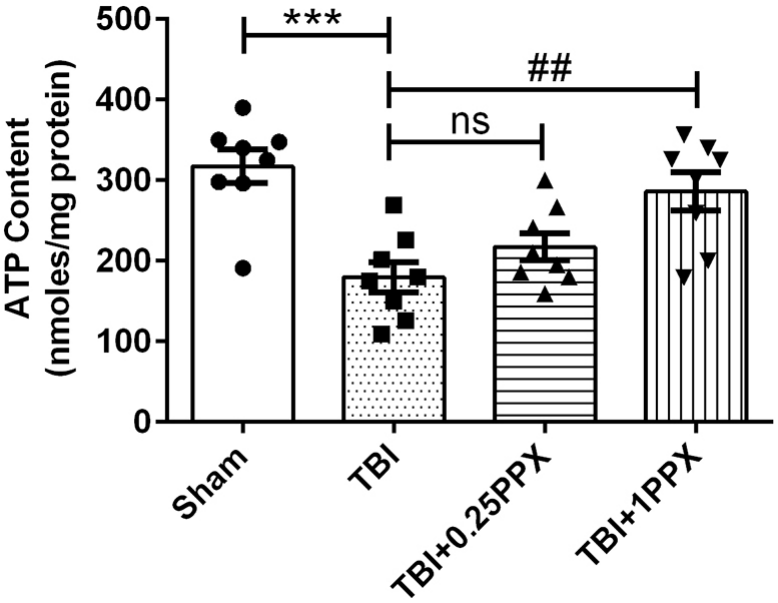
**PPX administration modulates ATP content levels.** ATP content was significantly decreased in the TBI rats compared with the sham rats. PPX treatment increased the level of ATP after a TBI, significantly at 1.0 mg/kg b.wt. ***P<0.001 compared with the sham group; ^##^*P*<0.01 compared with the TBI group; ns, not significant (one-way ANOVA with Tukey's multiple comparisons test). Data are presented as mean±s.e.m. (*n*=8).

Further, we investigated adenosine triphosphate (ATP) content in the experimental rats' brain tissue. We observed a significant decrease [*F*_(3,28)_=9.74, *P*<0.0001] in ATP content in the TBI rats compared with the sham rats. Interestingly, 1.0 mg/kg b.wt. PPX administration increased (*P*<0.01) ATP content among the TBI rats, but there was no significant change in ATP content with a low dose of PPX (Fig. 7).

### PPX treatment activates the Nrf2/HO-1 signaling pathway in TBI rats

**Fig. 8.**
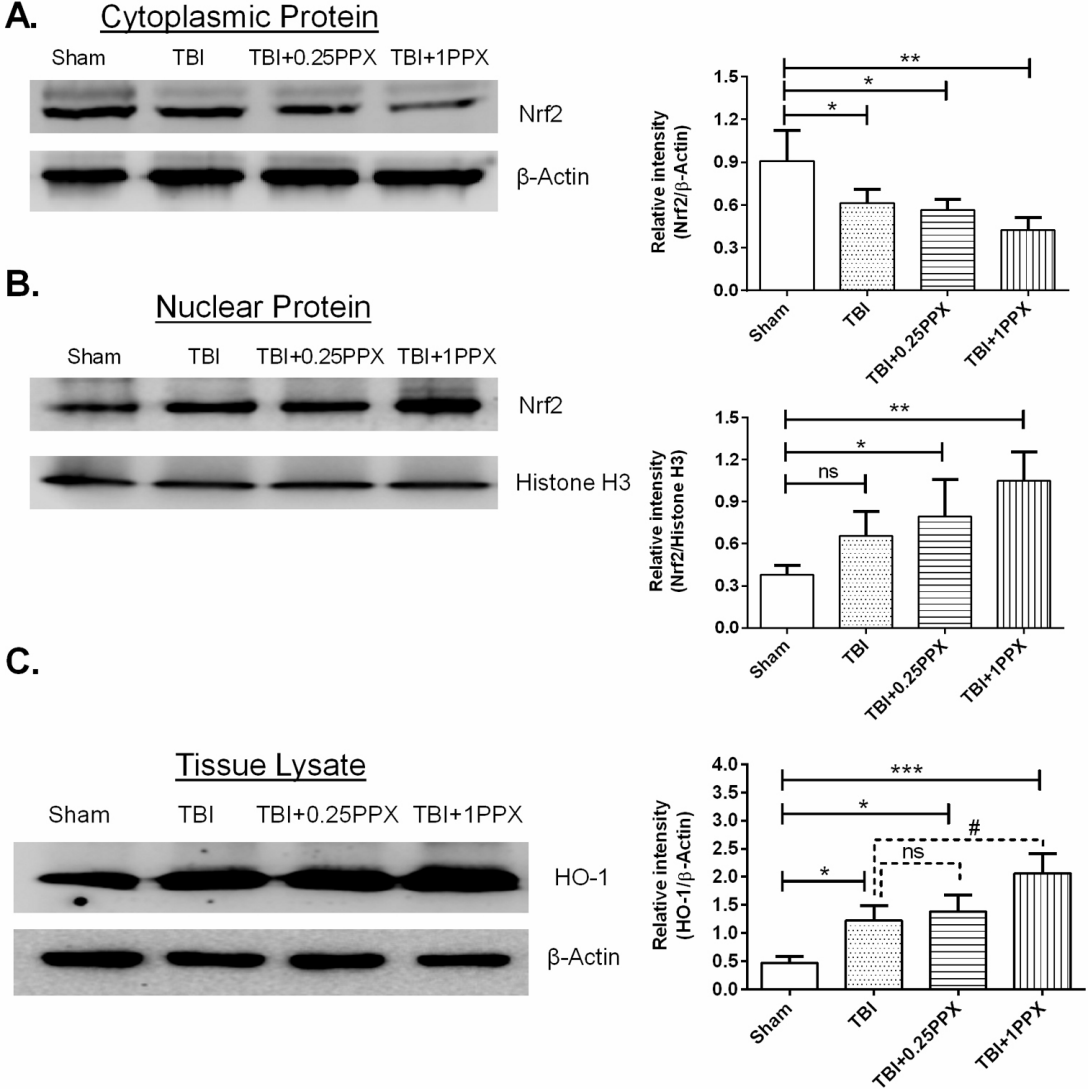
**PPX administration accelerates the translocation of Nrf2 from the cytoplasm to the nucleus and increases the expression of HO-1 after a TBI.** (A-C) Representative immunoblot intensity indicates cytosolic and nuclear Nrf2 (A,B), and HO-1 in the tissue lysate (C). The intensity of the targeted protein bands was normalized to β-actin and histone H3. ***P<0.001, **P<0.01 and *P<0.05 compared with the sham group; ^#^*P*<0.05 compared with the TBI group; ns, not significant (one-way ANOVA with Tukey's multiple comparisons test). Data are presented as mean±s.d. (*n*=4).

Western blot analysis was applied to detect the expression level of Nrf2 protein in nuclear as well as cytosolic fractions. As shown in Fig. 8, these results revealed that PPX treatment (0.25 mg/kg b.wt. and 1.0 mg/kg b.wt.) promoted Nrf2 nuclear translocation (*P*<0.05 and *P*<0.01, respectively) in the TBI rats compared with the sham rats. Additionally, a significant decrease in cytosolic Nrf2 was observed in the TBI and PPX-treated rats (*P*<0.05 and *P*<0.01, respectively), compared with the sham rats. The expression of HO-1 increased significantly following TBI (*P*<0.05). PPX post-treatment further increased HO-1 protein levels [0.25 mg/kg b.wt. (*P*<0.05) and 1.0 mg/kg b.wt. (*P*<0.001)] in TBI rats compared with the sham rats. When we compared the expression of HO-1 in the PPX-treated groups and TBI only group, we observed that PPX post-treatment increased the expression of HO-1 after a TBI.

### PPX treatment attenuates histological deficits in TBI rats

To identify histological deficits, we performed Nissl staining of Nissl bodies in neurons and calculated neuronal survival in the perilesional area of the cortex after TBI and PPX post-treatment. Microscopic examination of coronal brain sections showed normal neurons, as indicated by round shape and light-purple/blue color, and damaged neurons, indicated by cell shrinkage and dark staining. The TBI rats had a significantly reduced number of intact neurons (*P*<0.01) compared with the sham rats. Additionally, PPX (1.0 mg/kg b.wt.)-treated rats had a significantly higher number of intact neurons (*P*<0.05) compared with the TBI only rats. No significant changes were observed at a low dose of PPX (Fig. 1B).

## DISCUSSION

Specific to traumatic brain injuries, the primary injury generates oxidants, and the derivatives play a critical role in the progression of secondary injury damage, leading to organelle dysfunction and neuronal cell death (Zhang et al., 2017b). In this study, we examined the neuroprotective properties of PPX post-treatment in a controlled cortical impact model of TBI in Wistar rats. We found that PPX treatment ameliorated oxidative damage, and mitochondrial and behavioral dysfunction through mitochondria-mediated neuroprotection. In addition, PPX led to a protective effect via activation of the antioxidant Nrf2-ARE pathway, including by reducing LPO rate, increasing GSH levels, increasing GPx and SOD1 activities, decreasing 4-HNE levels, increasing HO-1 expression, decreasing the Bax/Bcl2 ratio and decreasing cyt-c release, as seen in the TBI-subjected rats. Therefore, the major findings of the present study are that PPX supplementation significantly reduced ROS formation, increased Δψm and ATP levels, and reduced mitochondrial-induced apoptosis in TBI rats owing to its antioxidant and anti-apoptosis properties. To the best of our knowledge, this is the first study to evaluate the mechanism underlying mitochondrial-targeted PPX neuroprotection following a TBI.

During the past few decades, a number of research articles have demonstrated that TBI induces a series of behavioral impairments in experimental rodents and traumatized human patients by damaging brain tissue and causing neuronal loss (Wu et al., 2016; Hoane et al., 2008). In the present study, TBI-induced neurological deficits were evidenced by the performance of TBI-subjected rats in grip, rotarod and beam balance tests. Our results indicated improved behavioral function among the TBI-inflicted rats following the administration of PPX. In addition, low-dose PPX resulted in behavioral improvement as reported in another neurodegenerative model (Ma et al., 2016; Andrabi et al., 2019). However, no significant changes were observed at the molecular level in our TBI model. Our findings are, therefore, in agreement with previous research on the neuroprotective effects of other therapeutic agents following a TBI (Ni et al., 2019).

It has been reported that two types of injuries are typically associated with TBI: mechanical injury, which is an irreversible injury, and secondary injury (or non-mechanical injury), which is reversible. The secondary injury begins after the primary injury, regulates various cascades, and activates neuronal apoptosis and dysfunction (Zhang et al., 2018). Oxidative stress is mainly caused by secondary injury, which plays a contributing role in the oxidation of macromolecules and neuronal dysfunction following TBI pathology. Additionally, excess free radical generation causes increased peroxidation of lipids and proteins, producing active carbonyls, aldehydes and ketones, including malondialdehyde (MDA), 4-HNE and acrolein (Ayala et al., 2014; Barrera et al., 2018). 4-HNE is a toxic aldehydic by-product of LPO and a biomarker of oxidative stress. Increased levels of 4-HNE indicate more serious oxidative damage in TBI pathogenesis as reported by Krishna et al. (2019).

During normal conditions, antioxidant enzymes such as SOD1 and GPx can inhibit oxygen free radical generation and protect the brain tissue against oxidative damage. In the present study, we found that TBI significantly elevated LPO rate and 4-HNE levels, and reduced GSH levels and the activities of SOD1 and GPx. We demonstrate, for the first time, that PPX significantly reduced LPO rate and 4-HNE, and significantly increased the activities of SOD1 and GPx antioxidant enzymes. Thus, our study results reveal that PPX ameliorates oxidative damage induced by TBI.

Loss of mitochondrial function has long been linked with the onset of neurodegenerative pathologies, such as Alzheimer's disease, Parkinson's disease, stroke (Cabral-Costa and Kowaltowski, 2019) and TBI (Lamade et al., 2019). The pharmacological agents that can attenuate these pathologies via mitochondrial-mediated neuroprotection are considered potential tools for treating neuronal death involving mitochondrial pathology. Previously, we have demonstrated that PPX induces protective effects against the mitochondrial pathway by closing mtPTPs (Andrabi et al., 2019). In the current study, we found that PPX post-treatment at high dose (1.0 mg/kg b.wt.) attenuated behavioral dysfunction and oxidative damage through mitochondrial-mediated neuroprotective effects and activation of the Nrf2/HO-1 pathway. It has been demonstrated that TBI induces excess ROS/RNS production, leading, in turn, to detrimental effects on lipids, proteins, nucleic acids and organelle function (such as that of mitochondria), progression of neuronal cell death and possible behavioral deficits (Angeloni et al., 2015). Excess ROS production could lead to oxidative damage and depletion of the Δψm, which can cause activation of cascades leading to mitochondrial damage and cyt-c release, and, in turn, promote mitochondrial-mediated neuronal cell death after TBI. In our study, TBI rats demonstrated a marked increase in ROS production and depletion of Δψm compared with the sham rats. PPX treatment in TBI rats attenuated ROS production, restored the integrity of Δψm and ATP generation, and provided neuroprotection from oxidative damage and mitochondrial dysfunction. Thus, the present findings are consistent with previous findings that PPX post-treatment induces neuroprotection via a mitochondrial pathway (Boscolo et al., 2013; Sayeed et al., 2006).

Bcl2 family-related proteins [Bcl2, Bcl-xL (also known as Bcl2l1), Bax and Bak1] play an essential role in the neuronal apoptotic pathway in various neurological diseases. Bax is a pro-apoptotic protein that is involved in TBI-induced apoptosis in the brain, whereas Bcl2 is an anti-apoptotic protein and a strong neuronal death suppressor (Raghupathi et al., 2000). Increased expression of Bax and decreased expression of Bcl2 often occurs during the progress of neuronal injury after a TBI (Strauss et al., 2004). The translocation of Bax from the cytosol to the mitochondrial outer membrane may lead to release of cyt-c, which subsequently binds to apoptosis-inducing factors and promotes apoptosis. We determined the Bax/Bcl2 ratio, which predicts the apoptotic tendencies of neurons. Post TBI, there was a significant increase in Bax expression and a decrease in Bcl2 expression among the TBI-subjected rats. PPX administration significantly reduced the Bax/Bcl2 ratio. Our results are, thus, consistent with a previous report that PPX activates the PI3K/AKT/GSK-3β pathway, which in turn inhibits Bax upregulation and translocation (Ma et al., 2016). We also determined cytosolic cyt-c levels as a marker of mtPTP activation and mitochondrial-mediated cell death following TBI. Mitochondrial perturbation led to cyt-c release into the cytoplasm, which, in turn, activated apoptosis-inducing cascades and further activation of intrinsic apoptosis pathways. Post-treatment with PPX reduced the release of cyt-c by attenuating the mitochondrial damage. Previous studies have demonstrated that PPX inhibits cyt-c leakage in different cell lines and *in vivo* (Shin et al., 2007; Wang et al., 2018). Based on the present results, we can conclude that PPX treatment protects mitochondria by multiple pathways in TBI.

Excess free radical production can not only modulate oxidation of macromolecules but also initiate apoptosis as single molecules (Rigoulet et al., 2011). There are many pathways that can inhibit or scavenge free radicals. Further, they have the potential to attenuate various neuropathological conditions caused by oxidative damage. It has been documented that the Nrf2-ARE pathway is an endogenous cytoprotective molecular mechanism and provides neuroprotection against oxidative stress induced by TBI (Li et al., 2016) and other acute brain injuries (Chu et al., 2019). Nrf2 is a transcription factor that is associated with Kelch-like ECH-associated protein-1 (Keap1) in the cytosol. During stress or other pathological conditions, Nrf2 dissociates from Keap1 and translocates to the nucleus, where it activates various antioxidant genes and phase II enzymes such as HO-1 and NQO-1 by binding with an ARE, and reduces the oxidative stress and inflammatory response involved in the acute brain injury following a TBI (Li et al., 2019b).

Few studies have demonstrated the relationship between PPX and Nrf2 following brain injury. Recently, Wang et al. (2018) reported that PPX administration increased the expression of Nrf2 in 1-methyl-4-phenyl-1,2,3,6-tetrahydropyridine (MPTP)-induced Parkinson's disease. However, the molecular mechanism underlying the effects of PPX in TBI is poorly understood. The current study examined whether or not the Nrf2/HO-1 signaling pathway is involved in the neuroprotection provided by PPX after a TBI, and investigated the molecular mechanism by which PPX modulates the Nrf2/HO-1 signaling pathway following a TBI. Our data suggest that Nrf2 expression is upregulated in the nucleus after TBI and that PPX treatment (0.25 mg/kg b.wt. and 1.0 mg/kg b.wt.) greatly accelerates Nrf2 translocation into the nucleus. The expression of Nrf2 in the cytosol was significantly decreased in TBI and PPX-treated TBI rats compared with the sham rats. HO-1 is one of the most abundant enzymes regulated by Nrf2, and exerts antioxidant, anti-inflammatory and anti-apoptotic effects in different *in vitro* and *in vivo* models (Ndisang, 2017). We found that HO-1 expression was significantly increased in the TBI-subjected rats, and PPX treatment further increased HO-1 expression in comparison to that of the sham rats. Thus, our results support earlier findings by other research groups (Xia et al., 2019; Li et al., 2019a). Our findings provide evidence on the therapeutic potential of PPX for the treatment of oxidative damage and mitochondrial injury after trauma by non-dopaminergic mechanisms. However, additional studies are needed to better understand the precise mechanism underlying the neuroprotective effects of PPX for it to be used as a therapeutic for TBI.

It is important to acknowledge some of the limitations of the present work. First, we showed the protective effects of PPX at an early time point, and further research needs to be conducted to evaluate the effects of subchronic and chronic exposure to PPX following TBI. In addition, the remedial time point, optimal dosage and other drug administration routes of PPX treatment in acute TBI need to be addressed. The potential role of the dopamine pathway after PPX treatment following a TBI should also be investigated.

In conclusion, we here show in Wistar rats that PPX exerts significant neuroprotection against TBI-induced oxidative damage by modulating the Nrf2/HO-1 signaling pathway. Our findings also indicate that PPX treatment significantly improves mitochondrial function by attenuating mitochondrial damage after TBI. Overall, the neuroprotective effects of PPX suggest that it represents an alternative therapeutic candidate for treatment of TBI-induced pathology.

## MATERIALS AND METHODS

### Animals

Male Wistar rats (250-300 g), obtained from the Central Animal House Facility of Jamia Hamdard, New Delhi, India, were used in the present study. The Institutional Animal Ethics Committee (IAEC) of Jamia Hamdard approved all animal experiments (Registration No. 173/GO/ReBi/S/2000/CPCSEA; Project No. 1376). Experimental rats were kept in an isolated environment in a 12 h light-dark cycle with free access to filtered water and food *ad libitum*. All experiments were performed in accordance with the IAEC of Jamia Hamdard.

### Experimental design

The experimental animals were randomly divided into four groups: sham, TBI, TBI+0.25PPX [0.25 mg/kg b.wt., interperitoneally (i.p.)] and TBI+1PPX (1.0 mg/kg b.wt., i.p.). Please refer to Table S1 for further details on the numbers of animals in experimental groups. Animals were sacrificed by cervical dislocation after deep anesthetization with an overdose of ketamine and xylazine. The damaged brain tissue area was isolated for analysis of biochemical and molecular assessment at 48 h after TBI.

### TBI model

TBI was induced by a controlled cortical impact device with minor modifications in our laboratory (Zhang et al., 2018; Edward Dixon et al., 1991). In brief, the experimental rats were anesthetized (75 mg/kg b.wt. ketamine and 10 mg/kg b.wt. xylazine, i.p.) and mounted on a stereotaxic frame (David Kopf Instrument, Tujunga, CA, USA) with ear bars and an incision bar. A 6-mm diameter craniotomy was created on the right side of the skull by a trephine drill, without disturbing the dura. The craniotomy was located between the bregma and the lambda, 1 mm lateral from the midline. The cortical impact injury was performed with a 4-mm diameter tip (2 mm depth, 3 m/s velocity and 120 ms dwell time) for the brain injury. The sham group underwent the same surgical procedure, without induction of injury. After surgical procedures and recovery from anesthesia, animals were returned to their home cages and kept at an ambient temperature.

### Postoperative care

The experimental animals were kept in an isolated chamber at 22±3°C in individual cages (two animals per cage). All experimental rats received 0.9% saline (intravenously) to prevent dehydration, and included administration of antibiotic [Cefotaxime 10 mg/kg b.wt., subcutaneously (s.c.)] and analgesic (Buprenorphine-SR 0.05 mg/kg b.wt., s.c.). Standard rodent pellet diet and filtered RO water were kept in the cages throughout the 48-h experimental period so that the experimental animals could easily access it without any physical disturbance due to the craniotomy.

### PPX treatment

PPX (#A-1237; Sigma-Aldrich), at doses of 0.25 mg/kg b.wt. and 1.0 mg/kg b.wt., was dissolved in 0.9% sterile normal saline and administrated by intraperitoneal injection with repeated doses at 30 min, 8 h, 16 h, 24 h, 32 h 40 h and 48 h following TBI.

### Behavioral analysis

#### Rotarod test of motor coordination

Motor coordination was evaluated by Omnitech rotarod apparatus (Omnitech, Columbus, OH, USA) as described previously (Rasheed et al., 2018). Experimental animals were kept on a rotating rod at a constant speed (10 rpm). Their performance was evaluated as the maximum time spent on the rotating rod. The apparatus automatically recorded the time in ms. The mean score of three successive trials was calculated for each group and the maximum cut-off time was 1800 ms.

#### Grip test of muscular strength

The grip strength test was performed as described previously (Rasheed et al., 2018). A 50-cm-long string wire was pulled between two solid objects at 40 cm height. The experimental rats were placed on the string and evaluated according to the following scores: 0, falls off; 1, hangs onto string by two forepaws; 2, hangs onto string by two forepaws but tries to climb on string; 3, hangs onto string by forepaws; 4, hangs onto string by forepaws plus tail wrapped around the string; 5, escapes by crossing the string.

#### Beam balance test of motor impairment

A narrow beam walk was used to analyze the motor impairment of rats as described previously (Rasheed et al., 2018). Rats were placed on a narrow black wooden beam and allowed to traverse from one side to the other. The final test consisted of three trials in each task and the time taken by each rat to travel the total distance across the beam was calculated.

### Biochemical evaluation

#### Tissue preparation and mitochondria isolation

Animals were euthanized, and the injured tissue was carefully dissected (including pericontusional brain tissue located 2 mm from the margin of the contusion site in TBI rats) from the ipsilateral hemisphere using an adult rat brain slicer matrix (BSRAA002, Zivic Instruments, Pittsburgh, PA, USA) for biochemical and molecular analysis. Brain tissue from the same location was isolated in the sham group rats. Tissue was homogenized in ice-cold 0.1 M phosphate buffer (pH 7.4) to obtain 10% homogenate (w/v) and centrifuged at 21,380 ***g*** for 20 min at 4°C to obtain post-mitochondrial supernatant (PMS). Mitochondria were isolated by centrifugation using the methods of Waseem et al. (2016). In brief, an isolated brain sample was homogenized using a Polytron homogenizer in ice-cold buffer A, pH 7.5, containing 250 mM sucrose, 10 mM HEPES, 1 mM EGTA, and 0.1% fat-free bovine serum albumin (BSA) to obtain 10% homogenate (w/v). The homogenate was centrifuged at 1000 ***g*** for 10 min at 4°C, then the supernatant was collected and again centrifuged at 10,000 ***g*** for 10 min at 4°C. Thereafter, the pellet was collected and resuspended in ice-cold buffer B, containing 250 mM sucrose, 10 mM HEPES and 0.1 mM EGTA, pH 7.4, then centrifuged at 12,300 ***g*** for 10 min at 4°C. The pellet was resuspended in buffer B and centrifuged at 12,300 ***g*** for 15 min. The final obtained pellet was dissolved in buffer C, pH 7.4, containing 250 mM sucrose, 10 mM HEPES and 0.1% fat-free BSA, and centrifuged at 12,300 ***g*** for 20 min at 4°C. The final pellet was resuspended in buffer C. Biochemical parameters were performed on the same day after the isolation of mitochondria.

### Assessment of oxidative damage

#### LPO

LPO was assessed in the injured ipsilateral tissue as well as isolated mitochondria according to the procedure of Andrabi et al. (2017). Briefly, 200 μl isolated brain mitochondria and brain homogenate were mixed individually with an incubation solution [10 μl of 10 mM butylated hydroxytoluene (BHT), 3 ml of 1% orthophosphoric acid (OPA) and 1 ml of 0.67% thiobarbituric acid (TBA)] and the mixture was incubated at 90°C for 45 min in a water bath. After cooling, the mixture was centrifuged in 2-ml Eppendorfs at 1080 ***g*** for 10 min at 4°C, and absorbance was measured at 535 nm. The rate of LPO was measured as µmol/nmol TBARS formed/h/g tissue or mg protein using a molar extinction coefficient (MEC) 1.56×10^5^ M^−1^ cm^−1^.

#### Reduced GSH levels

GSH levels were measured using a reduced GSH colorimetric assay kit according to the manufacturer’s guidelines (#E-BC-K030-S, Elabscience). Tissue homogenate (5%, w/v) was centrifuged at 10,000 ***g*** at 4°C. A supernatant was used for further analysis to determine the GSH content. For mitochondrial GSH content, mitochondrial samples (300 µg protein) were incubated in 500 µl of 0.1 M/l phosphate buffer (pH 7.5) for 10 min at 4°C, and further steps were performed according to manufacturer guidelines. The results were expressed as GSH activity (ng GSH/mg protein).

#### GPx activity

GPx activity was measured according to the procedure of Chaudhary and Parvez (2012). The reaction mixture contained 100 µl PMS, 0.1 M phosphate buffer, 1 mM EDTA, 1 mM sodium azide, 1 mM GSH, 0.2 mM NADPH and 0.25 mM H_2_O_2_. Oxidation of NADPH was measured at 340 nm. The enzyme activity was expressed as nmol NADPH oxidized/min/mg protein using the MEC 6.22×10^3^ M^−1^ cm^−1^.

#### SOD1 activity

SOD1 activity was measured using the methods of Chaudhary and Parvez (2012) with some modifications. The reaction mixture contained 0.8 ml glycine buffer (pH 10.4, 50 mM), 180 μl brain tissue sample prepared in a glycine-glycine buffer and 20 μl epinephrine in a 1-ml cuvette. SOD1 activity was measured kinetically at 480 nm as µmol epinephrine protected from oxidation/min/mg protein by using the MEC 4020 M^−1^ cm^−1^.

#### Assessment of mitochondrial ROS production

Mitochondrial ROS production was analyzed by flow cytometry analysis using di-chloro, di-hydro-fluorescein-diacetate (DCFDA) dye and the methods of Andrabi et al. (2017). Briefly, isolated mitochondria samples were diluted in the analysis buffer and incubated with 10 μM DCFDA dye for 5 min at 37°C in the dark. The flow cytometric acquisition of MFI from 10,000 events was made using a BD-LSR II (BD Biosciences) and histograms were generated using FACS-DIVA analysis software.

#### Assessment of Δψm

Δψm was measured by flow cytometry analysis using TMRE dye and the methods of Andrabi et al. (2017). Briefly, the isolated brain mitochondria sample was diluted in an analysis buffer and incubated with 0.1 μM TMRE dye for 15 min at 37°C in the dark. The flow cytometric acquisition of MFI from 10,000 events was made using the BD-LSR II, and histograms were generated using the FACS-DIVA analysis software.

### ATP analysis

ATP content was measured using an ATP assay kit (Elabscience, #E-BC-K157-S) according to the manufacturer’s guidelines. Brain tissue was homogenized in double-distilled water and incubated in a water bath at 90°C for 30 min. After incubation, the homogenate was centrifuged at 10,000 ***g*** for 10 min, and then the supernatant was mixed with substrates and transferred into 96-well plates for colorimetric analysis.

### Western blotting

At 48 h after TBI, injured right ipsilateral brain sample was homogenized in lysis buffer in the presence of protease and phosphatase inhibitor cocktail as reported by Sanderson et al. (2013) with some modifications. In brief, the lysed brain tissue mixture was centrifuged at 21,300 ***g*** for 30 min at 4°C. After centrifugation, the supernatant was resolved by 10-15% sodium dodecyl sulfate-polyacrylamide gel electrophoresis (SDS-PAGE) and then transferred to polyvinylidene fluoride (PVDF) membranes. Subcellular fraction isolation was performed using fractionation kits (BioVision, USA) according to the manufacturer’s guidelines. The protein bolts were blocked with 5% donkey serum. After blocking, blots were incubated with primary antibodies, including those against Bcl2 (1:1500; ABM0010, Abbkine), cyt-c (1:500; 136F3, Cell Signaling Technology), 4-HNE (1:1000; ab46545, Abcam), β-actin (1:2000; A01010, Abbkine), Bax (1:1000; sc-439, Santa Cruz Biotechnology), Nrf2 (1:500; E-AB-32280, Elabscience), histone H3 (1:2000; D1H2, Cell Signaling Technology), HO-1 (1:1000; GTX101147, GeneTex) and COX-IV (1:1000, #GTX114330, GeneTex) overnight at 4°C. The next day, bolts were washed with PBST (phosphate buffered saline, pH 7.5, with 0.025% Tween 20) and incubated with appropriate horseradish peroxidase-conjugated secondary antibodies (1:10,000; anti-rabbit, Invitrogen; anti-mouse, Abbkine) for 90 min at room temperature. The level of protein expression was visualized by enhanced chemiluminescence. Immunoblot densitometry analysis was performed using ImageJ version 1.50.

### Nissl staining

For Nissl staining, coronal sections were stained with 1% Cresyl Violet (C5042, Sigma-Aldrich) staining solution for 30 min at room temperature. Then, brain sections were washed with regular tap water and a dehydration procedure was performed using ethanol grades (30, 50, 70, 90 and 100%) for 5 min at room temperature, before fixing with DPX (Sigma-Aldrich) mounting medium. Brain sections were observed under an inverted Zeiss microscope. The number of intact neurons was counted per mm^2^ in each picture area of the cortex and expressed as the mean of three different brain sections for each group of rats.

### Data analysis

All results from the present study were collected by experimenters blinded to treatment conditions and results were reported as the mean±s.d. for immunoblots. For behavioral and biochemical analyses, values were expressed as the mean±s.e.m. Comparisons among multiple groups were analyzed using one-way ANOVA followed by Tukey's post hoc test, and statistical analysis was performed using Prism software version 6 (GraphPad Software, San Diego, CA, USA). Significant differences were defined as *P*-value <0.05.

## Supplementary Material

Supplementary information
