# Supplementary material for: Nrf2/HO-1 mediates the neuroprotective effects of pramipexole by attenuating oxidative damage and mitochondrial perturbation after traumatic brain injury in rats
Source: Dis Model Mech. 2020 Aug 19;13(8):dmm045021. doi: 10.1242/dmm.045021 (PMC7449795; doi:10.1242/dmm.045021)
Supplement: Supplementary information [file dmm-13-045021-s1.pdf]

Table S1. Number of animals used in each group.

<table>
<tr><th rowspan="2">Experiments</th><th colspan="4">Experimental Groups</th></tr>
<tr><th>Sham (Control)
(No. of Animals)</th><th>TBI
(No. of Animals)</th><th>TBI+0.25PPX
(No. of Animals)</th><th>TBI+1.0PPX
(No. of Animals)</th></tr>
<tr><td>Behavioral</td><td rowspan="2">8</td><td rowspan="2">8</td><td rowspan="2">8</td><td rowspan="2">8</td></tr>
<tr><td>Biochemical Analysis</td></tr>
<tr><td>Western Blot</td><td>4</td><td>4</td><td>4</td><td>4</td></tr>
<tr><td>Mitochondrial membrane potential</td><td rowspan="3">8</td><td rowspan="3">8</td><td rowspan="3">8</td><td rowspan="3">8</td></tr>
<tr><td>ROS</td></tr>
<tr><td>ATP</td></tr>
<tr><td>Nissl Stain</td><td>3</td><td>3</td><td>3</td><td>3</td></tr>
</table>

Disease Models & Mechanisms • Supplementary information
